# Marked Increases in Continuous Glucose Monitor-Detected Hypoglycemia During a Seven-Day Water-Only Fast in Healthy Men and Women

**DOI:** 10.1177/19322968261421956

**Published:** 2026-02-22

**Authors:** Kristoffer J. Kolnes, Lauren V. Turner, Steffen Brufladt, Emelie T. F. Nilsen, Anders J. Kolnes, Stephen O’Rahilly, Jørgen Jensen, Michael C. Riddell

**Affiliations:** 1 Department of Physical Performance, Norwegian School of Sport Sciences, Oslo, Norway; 2 Steno Diabetes Centre Odense, Odense University Hospital, Odense, Denmark; 3 School of Kinesiology and Health Science, Muscle Health Research Centre, York University, Toronto, ON, Canada; 4 Section of Specialized Endocrinology, Department of Endocrinology, Oslo University Hospital, Oslo, Norway; 5 Faculty of Medicine, University of Oslo, Oslo, Norway; 6 MRC Metabolic Diseases Unit, Institute of Metabolic Science, University of Cambridge, Cambridge, UK

**Keywords:** CGM, exercise, fasting, hypoglycemia, metabolic regulation, OGTT

## Abstract

**Background:**

Multiday fasting is practiced globally for various health or religious reasons which can cause marked declines in circulating glucose levels. Yet, the extent of hypoglycemia exposure (ie, blood glucose <70 mg/dL), as measured by continuous glucose monitoring (CGM) during a prolonged fast is unclear. We aimed to determine the distribution of interstitial glucose data as measured by CGM, during a seven-day water-only fast in healthy men and women.

**Methods:**

This study used interstitial glucose levels from CGM (Dexcom G4 Platinum) to profile hypoglycemic exposure during a seven-day water-only fast in 12 healthy adults (seven men; age 29.7 ± 6.1 years; body mass index [BMI] 25.0 ± 3.3 kg/m²) that also included physical performance tests (day 6) and an oral glucose tolerance test (day 7).

**Results:**

Time <70 mg/dL increased from 3.0% ± 7.1% at baseline to 66.0% ± 25.7% by day 5 (*P* < .001). Minimum daily glucose levels also declined from 76 ± 14 mg/dL at baseline to 50 ± 7 mg/dL by day 5 (*P* < .001). The performance tests and the oral glucose tolerance test markedly increased glycemia. No symptoms of hypoglycemia were reported.

**Conclusions:**

This research demonstrated considerable hypoglycemia exposure occurs without symptoms in heathy men and women who undertake multiday fasting.

## Introduction

Voluntary fasting is practiced globally by healthy individuals and individuals living with chronic metabolic diseases such as obesity and/or diabetes.^1,2^ The motivations for prolonged fasting—sometimes with periods of fasting extending for several days—encompass religious observance as well as potential therapeutic benefits, such as enhanced metabolic regulation, increased longevity, and improved weight control.^2^ Beyond voluntary practices, periods of prolonged fasting or markedly reduced caloric intake may also occur in clinical settings, including during hospitalization,^3-5^ bariatric surgery,^6^ or anorexia nervosa,^7^ where nutritional intake may be restricted.

While prolonged water-only fasting is generally well-tolerated for 12 to 24 hours in people without diabetes, it has been associated with some hypoglycemia exposure, at least according to limited point-in-time sampling of glucose concentration either in whole blood or in plasma samples taken in laboratory or clinical settings.^8,9^ However, the extent of hypoglycemia exposure during prolonged fasting in a healthy population has not yet been quantified with the use of continuous glucose monitors (CGMs). This study examined the extent of hypoglycemic exposure using real time CGM during a seven-day fast that included performance tests (day 6) and an oral glucose tolerance (OGTT, day 7) test in healthy men and women.

## Materials and Methods

This manuscript expands upon findings initially reported on the effects of seven days fasting on physical performance and metabolic adaptations by Kolnes et al.^10^ In brief, 12 healthy adults (7 men, 5 women) with a mean ± standard deviation (SD) baseline age of 29.7 ± 6.1 years, body mass index (BMI) of 25.0 ± 3.3 kg/m^2^ and with a body fat content of 23.4% ± 6.9% completed a seven-day water-only fast while maintaining their typical daily routines (including physical activity and work) and wearing an blinded CGM (Dexcom G4 Platinum; Dexcom Inc, San Diego, CA, USA) with calibration each morning and evening (HemoCue Glucose 201 RT; Ängelholm, Sweden). Whole blood was also collected at baseline and each morning during the fast (6-9 a.m.). The study was conducted in accordance with the Declaration of Helsinki and was approved by the Ethics Committee at the Norwegian School of Sport Sciences (15-220817).

CGM data were averaged per-day within each participant from day −1 (Baseline) through day 7, with day 0 being the first day of fasting. At day 6, the physical performance tests included maximal isokinetic and isometric strength assessments (~7 min), maximal fat oxidation (~23 min) and maximal oxygen uptake assessment (~5-10 min), which increased plasma glucose level.^10^ The fast was broken at ~9am on day 7 with an OGTT, and data collection ended mid-day. Outcomes in this analysis included CGM-measured minimum, maximum, and mean interstitial glucose, percent (%) time in tight range (TITR: 70-140 mg/dL), % time <70 mg/dL, and % time >140 mg/dL. Mean glucose during the fasting period (days 0-5) was also averaged and compared between males and females. Any CGM value reported as “low” was imputed as 40 mg/dL, the device’s lower detection limit. Normality was assessed via the Shapiro-Wilk test, and daily CGM metrics were compared with baseline using a paired *t* test (normal distribution) or Wilcoxon-sign ranked test (nonnormal distribution). A repeated measures mixed effects analysis was used to determine overall between-group differences. Statistical analyses were conducted in GraphPad Prism version 10.4.2 (GraphPad Software, Boston, MA), with significance as *P* < .05.

## Results

**Figure 1.**
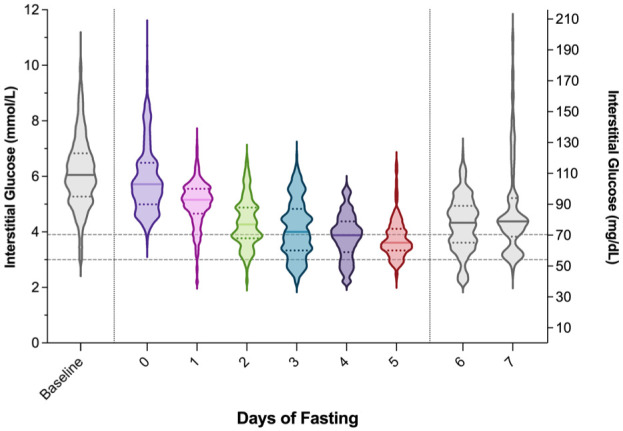
Continuous glucose monitoring (CGM) data distribution by days of fasting. Violin plots present the distribution of 24-hour CGM data from day −1 (baseline) through day 5, with day 6 (performance tests) and day 7 (oral glucose tolerance test) also shown. Plots extend from minimum to maximum values, with the horizontal dotted lines within each plot indicating the interquartile range with medians indicated by a solid horizontal line. Gray horizontal dotted lines indicate the thresholds for level 1 (<70 mg/dL) and level 2 (<54 mg/dL) hypoglycemia.

The distribution of CGM glucose values across the fasting period is shown in Figure 1. With increasing days of fasting, there was a progressive downward shift and narrowing of glucose values. Median (IQR) glucose decreased from 110 (95-123) mg/dL at baseline to 65 (59-74) mg/dL by day 5 of fasting, indicating both lower central values and reduced variability over time. When examining sex-based differences, mean ± SD glucose during the fast was not significantly different between males (83 ± 18 mg/dL) and females (77 ± 17 mg/dL, *P* = .065).

**Figure 2.**
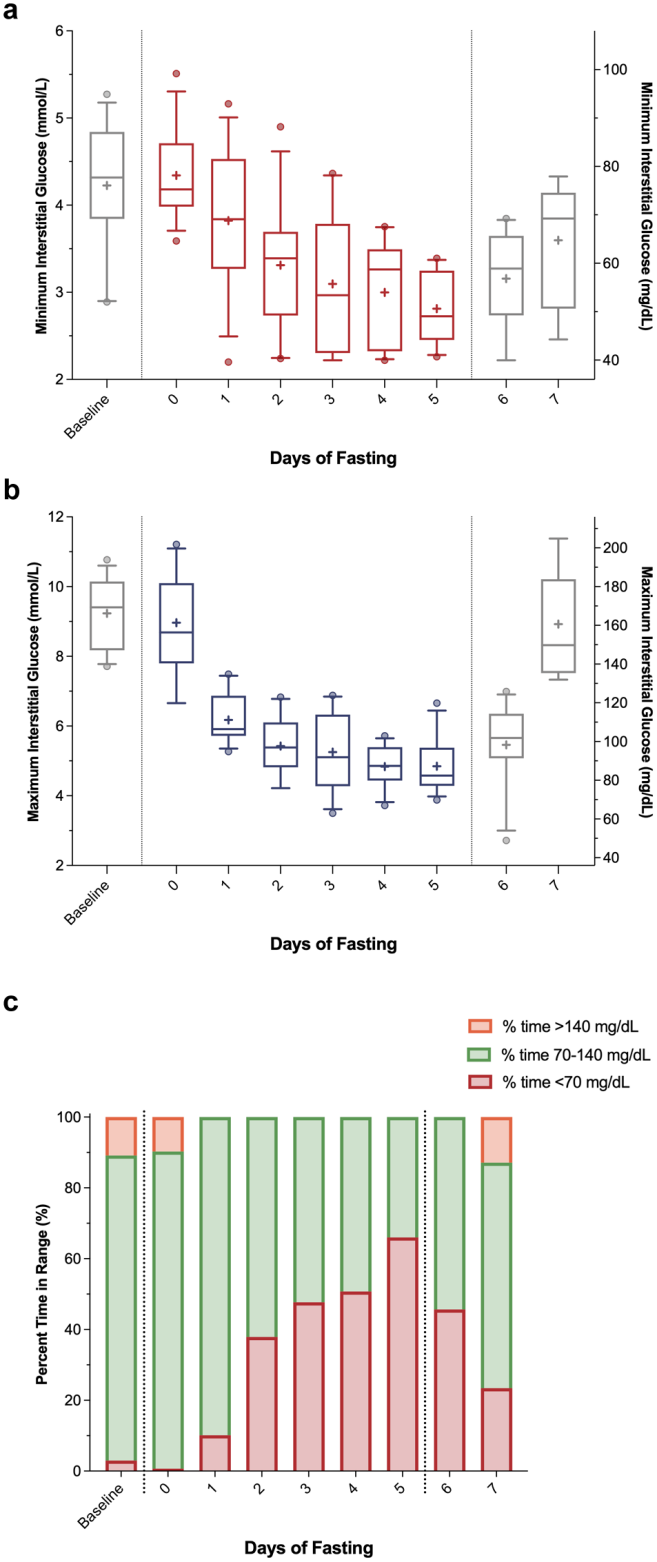
Continuous glucose monitoring (CGM) metrics by days of fasting from day −1 (baseline) through day 5, with day 6 (performance tests) and day 7 (oral glucose tolerance test). Box plots are presented for minimum (a) and maximum (b) measured daily interstitial glucose values. Boxes represent the 25th to 75th percentiles, whiskers extend from the 10th to 90th percentiles, the horizontal line within each box indicates the median, the plus sign denotes the mean, and outliers are shown as individual dots. Daily percent time in range metrics (c) highlight the proportion of time spent below range (<70 mg/dL, red), in tight range (70-140 mg/dL, green), and above range (>140 mg/dL, orange).

Minimum, maximum, and mean interstitial glucose values declined significantly across the entire seven-day period (Table 1, all *P* < .001). As shown in Figure 2a, minimum glucose decreased from 76 ± 14 mg/dL at baseline to 50 ± 7 mg/dL by day 5 (*P* < .001). Maximum glucose (Figure 2b) fell from 166 ± 20 mg/dL at baseline to 88 ± 14 mg/dL by day 5 (*P* < .001) and remained relatively suppressed on the day with the performance tests (99 ± 22 mg/dL, *P* < .001) but not on the OGTT day (160 ± 27 mg/dL, *P* > .05). Mean daily glucose followed a similar pattern, declining from 112 ± 14 mg/dL at baseline to 67 ± 7 mg/dL on day 5 (*P* < .001), but rising again with the performance tests (day 6) and OGTT (day 7) days. Significant changes % time in range metrics were also observed throughout the fasting period (Figure 2c). The % time <70 mg/dL increased progressively during the fast, rising from 3.0% ± 7.1% at baseline to a peak of 66.0% ± 25.7% on day 5 (*P* < .001). Simultaneously, % time >140 mg/dL was virtually eliminated while TITR declined from 86.2% ± 16.0% at baseline to 34.0% ± 25.7% on day 5 (*P* < .001).

**Table 1.** CGM Metrics Compared Among Study Days (N=12) From Day −1 (baseline) Through Day 5, With Day 6 (Performance Tests) and Day 7 (Oral Glucose Tolerance Test) Also Shown.

	Baseline	Day 0	Day 1	Day 2	Day 3	Day 4	Day 5	Day 6	Day 7	*P* -val^b^
% time <70 mg/dL	3.0 ± 7.1	0.7 ± 2.1^a^	10.2 ± 14.1^a †^	37.9 ± 28.7^†^	47.7 ± 37.2^†^	50.7 ± 33.4^‡^	66.0 ± 25.7^‡^	45.6 ± 37.9^†^	23.4 ± 31.8^a^	<.001
% time 70-140 mg/dL	86.2 ± 16.0	89.6 ± 14.5	89.9 ± 14.1	62.1 ± 28.7^†^	52.3 ± 37.2^†^	49.3 ± 33.4^†^	34.0 ± 25.7^‡^	54.4 ± 37.9*	63.7 ± 35.6*	.002
% time >140 mg/dL	10.9 ± 15.7	9.7 ± 14.6	0.0 ± 0.0^a ‡^	0.0 ± 0.0^a ‡^	0.0 ± 0.0^a ‡^	0.0 ± 0.0^a ‡^	0.0 ± 0.0^a ‡^	0.0 ± 0.0^a ‡^	12.9 ± 18.4^a^	.019
Min glucose (mg/dL)	76 ± 14	77 ± 9	68 ± 14	59 ± 14*	56 ± 14^†^	54 ± 11^†^	50 ± 7^‡^	58 ± 11^†^	65 ± 13	<.001
Max glucose (mg/dL)	166 ± 20	162 ± 27	112 ± 13^‡^	97 ± 14^‡^	95 ± 20^‡^	86 ± 11^‡^	88 ± 14^‡^	99 ± 22^‡^	160 ± 27	<.001
Mean glucose (mg/dL)	112 ± 14	106 ± 13	90 ± 9^‡^	77 ± 13^‡^	74 ± 14^‡^	70 ± 13^‡^	67 ± 7^‡^	74 ± 16^‡^	94 ± 18*	<.001

Two-tailed significance when compared with baseline determined by a paired-test unless otherwise stated. *P ≤ .05. ^†^*P* ≤ .01. ^‡^*P* ≤ .001.

a Two-tailed significance when compared with baseline determined by a Wilcoxon sign-ranked test.

b *P*-value is reported for overall among day differences using a repeated measures mixed-effects analysis (note: two participants were missing CGM data for OGTT day).

## Discussion

This study provides an examination of interstitial glucose measurements using CGM during a seven-day water-only fast in healthy adults. While previous studies have examined the metabolic and performance-related consequences of prolonged fasting,^10^ the present analysis is the first to quantify the extent and distribution of hypoglycemia using CGM in a healthy population. Our findings reveal that even in metabolically healthy individuals, prolonged fasting leads to a progressive decline in interstitial glucose concentrations, with a marked increase in time spent below 70 mg/dL, with as much as 65% of the time observed in this range .

In individuals with diabetes on glucose lowering medications (ie, insulin, sulfonylureas, and meglitinides), hypoglycemia as measured by blood or interstitial glucose readings is common. However, it is generally held that people without diabetes with intact hepatic function rarely experience fasting hypoglycemia because of preventative counter-regulatory mechanisms. Our findings reveal that if a multiday fast is observed, the daily glucose nadir, as measured by CGM, falls into the biochemical hypoglycemic range as defined by CGM consensus guidelines for individuals living with diabetes.^11^ Importantly, hypoglycemia identified by CGM may not necessarily equate to *clinical hypoglycemia*, which is typically defined as the presence of symptoms, counterregulatory failure, or the need for intervention.^12^ Moreover, CGM may overreport hypoglycemic exposure because of limited accuracy in the low glucose range.^13^ It should also be stressed that existing glycemic targets for hypoglycemia for people living with diabetes (<4% time per day <70 mg/dL) are based on thresholds associated with increased risk of acute symptoms and long-term complications.^12^ However, participants in this study experienced an increase in CGM-measured time <70 mg/dL from approximately 3% at baseline to over 65% by day 5 of the fast, without any reported symptoms. These findings suggest that if a real-time CGM is used during multiday fasting in people without diabetes, low glucose values should be expected.

This prolonged “asymptomatic” hypoglycemia also raises important questions about the appropriateness of applying conventional CGM hypoglycemia thresholds to individuals without diabetes, with other chronic conditions, and/or those engaged in fasting practices. For example, in individuals following Roux-En-Y gastric bariatric surgery, CGM data have shown that three-quarters of patients (n=40) had CGM values <55 mg/dL (3.05 mmol/L), however this may be due to hyperinsulinemia rather than fasting. Nevertheless, ~80% of events were asymptomatic^6^ and while time spent in hypoglycemia averaged ~1 hour/day, participants in our cohort spent an estimated 9 to 16 hours/day with CGM measured hypoglycemia during the latter days of fasting, albeit using a more conservative (<70 mg/dL) threshold. Similarly, patients with anorexia nervosa have been reported to spend up to 20.8% ± 3.9% of the day below 70 mg/dL (range: 0%-52%) without clinical symptoms.^7^ Thus, revised terminology may be needed to clearly distinguish between CGM-detected low glucose and clinical symptomatic hypoglycemia across diverse physiological and clinical contexts.

Violin plots of CGM data from this study further reveal a downward shift in glucose distribution below 70 mg/dL during fasting, with no readings exceeding 119 mg/dL on the fifth day of fasting. This narrowed glycemic range and reduced variability are consistent with other reports of metabolic adaptations to prolonged fasting, including decreased hepatic glucose production, suppressed insulin secretion, and increased reliance on gluconeogenesis and ketone body oxidation.^10,14,15^ Notably, elevations in glucose were still observed in response to controlled stimuli of performance tests and OGTT.

The rise in interstitial glucose concentrations with performance tests (day 6) was confirmed by significant increases in plasma glucose concentrations (from 68 ± 4 to 101 ± 4 mg/dL) with fasted performance tests in these same individuals, as previously reported.^10^ The reasons for this rise are likely multifactorial. First, after a seven-day fast, circulating insulin levels are markedly diminished while counterregulatory hormones (glucagon, cortisol and catecholamines) are elevated at baseline and increase further with exercise, increasing hepatic glucose output.^16^ Second, circulating free fatty acid levels are elevated ~three-fold after a seven-day fast and serve as a primary fuel for exercise.^10^ Moreover, the mitochondrial enzyme pyruvate dehydrogenase kinase-4 (PDK4) increases ~13-fold and increased pyruvate dehydrogenase phosphorylation with a seven-day fast which dramatically reduced carbohydrate oxidation during the performance tests.^10^ Importantly, PDK4 is required to prevent hypoglycemia during fasting.^17^ Overall, these hormonal and enzymatic regulatory changes likely diminish skeletal muscles’ ability to oxidize carbohydrates, preventing exercise-associated hypoglycemia in healthy humans without diabetes. If these physiologic adaptations can be mimicked for individuals with type 1 diabetes who are prone to exercise-associated hypoglycemia is unclear.

Despite its novelty and strengths, this study has some limitations. First, the small sample size which included only young, healthy, nonobese adults limits the generalizability of these results. Second, the present analysis did not directly link glycemic outcomes with symptomatic experience of the participants. Third, the CGM did not report values below 2.2 mmol/L (40 mg/dL), and values below this threshold were truncated. Finally, a direct assessment of Dexcom G4 Platinum accuracy in this population was not performed, as paired plasma or capillary glucose measurements were not collected at predefined intervals during the study. It should be noted, however, that mean fasting plasma glucose level (67 mg/dL, as measured from the antecubital vein blood draw on day 5)^10^ was similar to the CGM-derived mean glucose from that same day (67 ± 7 mg/dL). In addition, in the pivotal accuracy study of the G4 CGM, the mean absolute relative difference was 9.0% and improved after the first day of CGM wear, and hypoglycemia (<70 mg/dL) was detected accurately 91% of the time.^18^

In conclusion, this study highlights that a sustained water-fast in healthy individuals can lead to a significant and sustained drop in interstitial glucose, with frequent exposure beyond the conventional hypoglycemic range (<70 mg/dL). Further research is warranted to explore how these prolonged low-glucose states affect cognition, performance, and long-term health outcomes in healthy and clinical populations.
